# Developmental and Evolutionary History Affect Survival in Stressful Environments

**DOI:** 10.1371/journal.pone.0095174

**Published:** 2014-04-18

**Authors:** Gareth R. Hopkins, Edmund D. Brodie, Susannah S. French

**Affiliations:** Department of Biology and the Ecology Center, Utah State University, Logan, Utah, United States of America; Tuscia University, Italy

## Abstract

The world is increasingly impacted by a variety of stressors that have the potential to differentially influence life history stages of organisms. Organisms have evolved to cope with some stressors, while with others they have little capacity. It is thus important to understand the effects of both developmental and evolutionary history on survival in stressful environments. We present evidence of the effects of both developmental and evolutionary history on survival of a freshwater vertebrate, the rough-skinned newt (*Taricha granulosa*) in an osmotically stressful environment. We compared the survival of larvae in either NaCl or MgCl_2_ that were exposed to salinity either as larvae only or as embryos as well. Embryonic exposure to salinity led to greater mortality of newt larvae than larval exposure alone, and this reduced survival probability was strongly linked to the carry-over effect of stunted embryonic growth in salts. Larval survival was also dependent on the type of salt (NaCl or MgCl_2_) the larvae were exposed to, and was lowest in MgCl_2_, a widely-used chemical deicer that, unlike NaCl, amphibian larvae do not have an evolutionary history of regulating at high levels. Both developmental and evolutionary history are critical factors in determining survival in this stressful environment, a pattern that may have widespread implications for the survival of animals increasingly impacted by substances with which they have little evolutionary history.

## Introduction

Natural and anthropogenic stressors are commonplace throughout the environment. The ways in which stressors impact organisms, and their ability to successfully respond to these stressors is of paramount importance to our understanding of biological systems. For organisms with complex life cycles, the ability to respond to a given stressor may vary depending on life history stage, and there may be carry-over effects from one stage to the next [1] (see Table S1 in Supporting Information). However, organisms may or may not have an evolutionary history of regulating the stressor in question, and this may also affect their ability to effectively respond [2], [3]. We propose that both an organism's developmental history of exposure to a stressor (developmental history hypothesis) and its evolutionary history of regulating that stressor (evolutionary history hypothesis) play critical roles in the survival of organisms in stressful environments.

It has been suggested that the earlier in an organism's life history environmental stressors are experienced, the more severe the lasting consequences will be [4]–[6], and there is strong empirical evidence across animal taxa for this assertion (Table S1). This forms the basis of our developmental history hypothesis. In humans, for example, the environment of the womb can significantly affect an individual's chances of cardiac and other diseases later in life [5], [7], [8]. In birds, the temperature at which eggs are incubated can affect hatchling body composition, growth, immunocompetence and thermoregulatory ability [4], [9]. Developmental temperature also affects survival, growth and behavior of juvenile reptiles (e.g., [10]) (Table S1). Elevated CO_2_ as embryos results in decreased larval settlement success of sea urchins [11], and the ability of bryozoans to produce large, successful colonies is dependent on their embryonic experience and growth [12]. Thus, embryonic exposure to stressors can be critical to an animal's future fitness (Table S1).

Parsing critical life history stages, however, is not trivial, and many studies have given contradictory evidence for the developmental history hypothesis. For example, while multiple studies have shown that embryonic environment can significantly affect an individual's chances of success in later life (Table S1), others have shown that it is the larval or juvenile environment that has the greatest influence on survival, growth, or reproduction (e.g., [13], [14]). Still others have shown that while the embryonic environment has a significant role to play in later life, its effect may be dependent on the environment animals experience later in life (e.g., [15], [16], [17]). Experiments are often not designed to isolate the effects of environment on a specific life history stage from those of another (e.g., [18], [19]–[21]), and thus, consistent knowledge of the environmental and carry-over effects across multiple life history stages is lacking (but see [14], [17], [22], [23]).

While there is a strong empirical basis for the developmental history hypothesis (even with the conflicting evidence and limitations identified above), there is much less known regarding the evolutionary history hypothesis. Organisms in most habitats today face both natural stressors with which they have an evolutionary history, and thus evolved physiological mechanisms of regulating (e.g., CO_2_, temperature, NaCl), and novel stressors with which they do not have this same evolutionary history (e.g., pesticides, flame retardants, commercial non-NaCl-based deicing salts), and thus lack the physiological mechanisms to regulate. The effects of developmental history must therefore be placed in this environmental and evolutionary context. While many studies have documented the significant effects of unfamiliar substances such as pollutants on evolutionarily-naïve organisms (e.g., reviewed by [24] for amphibians), these cannot be directly compared to stressors with which the organism has an evolutionary history, and thus a means of regulating, as the nature of the two stressors is usually very different (i.e., comparing the effect of a herbicide with the effect of NaCl). At this point, we do not know how the potentially important effects of an organism's evolutionary history with a stressor may interact with its developmental history of exposure with the stressor.

To address these concerns, we tested the effects of developmental and evolutionary history on survival in stressful environments. We chose the rough-skinned newt (*Taricha granulosa* Skelton; Caudata: Salamandridae) as our model, an osmotically sensitive organism, and salinity as its stressor. Specifically, we tested the effects of both NaCl and MgCl_2_ on the post-hatching survival of newt larvae that had either been exposed to salt as both embryos and larvae or just as larvae. Salinity is an excellent stressor to use to test our two hypotheses, as it is a naturally occurring abiotic component of aquatic habitats, and is known to have significant carry-over effects from the embryonic to post-hatching life-stages in a variety of organisms, [15], [23], [25]–[27] (Table S1). We used salt concentrations that were within environmentally relevant limits of freshwater aquatic systems impacted by either natural (i.e., estuaries) or anthropogenic (i.e., road deicing salts) sources of salts [29], [30]. The two most common sources of salinity in North America today are two different salts, NaCl and MgCl_2_, only one of which most organisms have an evolutionary history of regulating. Sodium chloride (NaCl) is one of the most common osmolytes, and organisms have an evolutionary history of regulating this in a variety of habitats, whereas MgCl_2_ has not been identified as a common vertebrate osmolyte [31], and Mg^2+^ is not found in substantial concentrations in most freshwater habitats, nor the precipitation that feeds them (including in the newts' range) [32]. Therefore, animals do not have the same evolutionary history of physiological regulation of this ion. Nevertheless, MgCl_2_ is now the second most commonly used road deicer in North America (behind NaCl), and is used exclusively in some areas of the continent [33]. Thus, there is the potential that organisms will encounter MgCl_2_ in substantial quantities in their environment. We found that both salts caused significant developmental carry-over effects from the embryonic environment on larval survival, but that the salts differed in their effects on larval survival, according to the differential evolutionary history that amphibians have with regulating the two stressors. As more and more freshwater animals, mostly maladapted to salt, will be forced to cope with increasing salinization of their habitats due to the application of road deicing salts [34], [35]–[36], landscape modification and agricultural waste [37]–[40], and rising sea-levels [41]–[43], understanding the effects of both developmental and evolutionary history of salinity exposure will have important implications for both life history and evolutionary theory, as well as conservation efforts.

## Materials and Methods

### Ethics Statement

Adult rough-skinned newts (*Taricha granulosa*) (not an endangered or protected species) were collected by dip-net and hand from Soap Creek ponds (44°40′13.22″N, 123°16′39.65″W) under Oregon Department of Fish and Wildlife Scientific Taking Permit #062-11. Access to these ponds was granted by Joe Beatty, Oregon State University. The Utah State University Institutional Animal Care and Use Committee (IACUC) approved the collection and use of animals in this research, and all experimental protocols (approved protocol #1524). Animals were euthanized at the completion of experiments with MS-222, in accordance with the approved IACUC protocol (#1524).

### Experimental Procedure

As reported in a previous study ([44] for detailed methods on habitat, field collection, rearing eggs and preparing salt solutions), we reared eggs from 16 different gravid wild-caught female rough-skinned newts (*Taricha granulosa*) from a single, salt-naïve population from Benton County, Oregon, in a laboratory environmental control chamber at 7°C. This population is truly salt-naïve [44], being highly philopatric to freshwater ponds that are separated by hundreds of meters from small county roads that are not salted (Kendal Weeks, Oregon Department of Transportation Road Maintenance, personal communication; Kent Mahler, Benton County Road Maintenance, personally communication). While MgCl_2_ is widely used in Oregon as its exclusive deicer, it is also not applied to the nearest stretch of highway to these ponds, located over 4 km away (Kendal Weeks, Oregon Department of Transportation Road Maintenance, personal communication). See [44] for additional details on this habitat. Eggs from wild-caught females were randomized to one of six different salt treatments, made with laboratory grade NaCl (Thermo Fisher Scientific, Fair Lawn, NJ, USA), MgCl_2_ (Acros Organics, Fair Lawn, NJ, USA) and distilled water (Low NaCl, Low MgCl = 1.0 g/l Cl^−^; Medium NaCl, Medium MgCl_2_ = 1.5 g/l Cl^−^; High NaCl, High MgCl_2_ = 2.0 g/l Cl^−^) and a control (20% Holtfreter's Solution = 0.7 g/l Cl^−^ [45]). Those eggs that survived these treatments were used in the present experiment. At hatching, the size (total length) and developmental stage [46] of hatchlings were recorded (see [44] for full methods and results).

**Figure 1:**
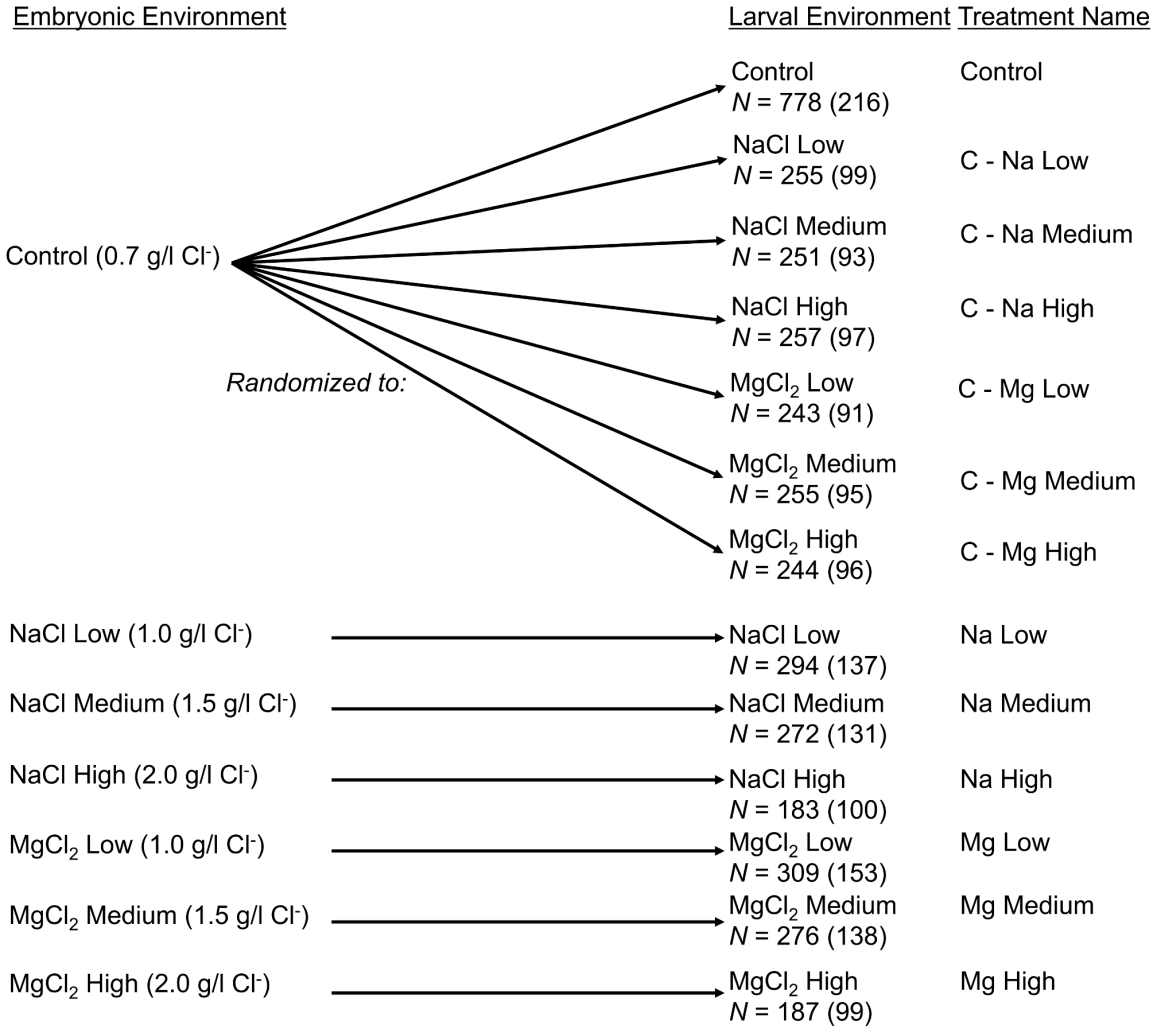
Outline of experimental design. Embryonic and larval environments, salinity concentrations, treatment names, and sample sizes are shown. Newt eggs were reared in either a freshwater control, or one of six salt treatments. Upon hatching, embryos that were reared in salt stayed in that salt, whereas embryos reared in control either stayed in control or were randomized to one of the six salt treatments for the larval environment. The name of each treatment combination is listed, and sample sizes are given under each larval environment (numbers outside of parentheses indicate total number of individuals in the treatment, whereas numbers inside parentheses indicate number of containers in the treatment (up to five sibling larvae were reared in the same container, and individuals within containers were treated as nested subsamples. See Methods for more details).

Eggs that were reared in a salt treatment remained in that salt treatment as larvae (Fig. 1). Approximately 7 times more control eggs were reared than salt treatment eggs, so that control eggs could be randomized to new larval treatments in the present experiment (similarly to [26]) (Fig. 1). Eggs were monitored daily and all larvae were transferred to their new treatment solution within 12 hours of hatching. This direct transfer, following a similar protocol of Petranka and Doyle [47], was meant to mimic the sharp spike in Cl^−^ concentrations found in road-side environments that immediately occurs within hours of a deicing event or snowmelt [48]–[50], where minimal to no time is allowed for acclimation. While gradual acclimation of low salinity levels have led to increased tolerance in some amphibians (e.g., [51]) it has also led to increased susceptibility in others [52], and is less environmentally relevant to examining the sudden spikes of salinity seen in habitats due to road deicing salt application. In addition, while the salt concentrations used were typical for those immediately resulting from deicing events [48], [53], they were also well below recorded NaCl and MgCl_2_ LD-50 values for other amphibian larvae [37], [54], [55].

Larvae were housed in sibling groups of up to 5 individuals (keeping offspring from different female and treatment combinations separate) in 12.5 cm diameter, 10.5 cm deep round plastic containers, filled with 400 ml of solution. Each container was randomized to a location in a growth chamber set at 7°C, with a 12 h light: dark photoperiod. Containers were checked daily for larval survival, and dehydration. Dead larvae were noted and removed, and a small amount of distilled water was added to each container if necessary, to compensate for evaporation. *Taricha granulosa* larvae retain some embryonic yolk for up to approximately two weeks after hatching, and do not engage in feeding on prey before then. As we did not want to confound our survival results with possible negative effects of the salt treatments on larval prey, we only conducted this experiment for 15 days post-hatching; if a larva was alive at day fifteen, it was recorded as alive for the purposes of the analysis. A similar endpoint has also been used in a previous study on post-hatching survival of frog larvae in road deicing salt [47].

### Statistical Analysis

For survival analyses, individual larvae were treated as subsamples within containers, which were treated as subsamples nested within individual female. Larval survival was analyzed using a binomial distribution, with a generalized linear mixed model blocking on individual female as a random effect. We first compared the survival of control newts (i.e., those reared in control as eggs and larvae) to newts in all other treatments for each salt, and then ran separate models to compare survival among salt treatments (minus control) for both larvae that were reared in control and those reared in salt as eggs, with Tukey-adjusted multiple comparisons among individual treatment levels, when an overall significant effect of treatment was found. We were, however, primarily interested in comparing and contrasting the effects of embryonic and larval environment on larval survival. As we did not have a complete factorial design in this study (e.g. embryonic low MgCl_2_ + larval high NaCl treatment combination), for this analysis, we analyzed the effects of the two different salt types separately, using embryonic and larval treatments as fixed effect factors in our models. We then analyzed the effect of embryonic versus larval environment on larval survival for each salt [56]. In these analyses, larval treatment had three levels, low, medium and high, and embryonic treatment had two levels, control and salt. This enabled a direct statistical comparison to be made of larval survival between animals that were reared as eggs in control or, for example, low MgCl_2_, for larvae that were reared in low MgCl_2_. We conducted Tukey-adjusted multiple comparisons, specifically comparing larval survival in each salt treatment level between eggs that were reared in either that salt treatment or control, for cases in which an overall significant effect of either embryonic treatment, larval treatment, or their interaction was found. Analyses were conducted using PROC GLIMMIX in SAS software version 9.3, with significance set at α = 0.05.

As embryonic exposure to salt affected the size and developmental stage at hatching of newts, as did differences among individual mothers (females) [44], we wanted to further assess the potential contribution of these variables, as well as embryonic and larval treatments in general, in explaining any overall effects of salt treatment in either embryonic or larval environments on larval survival. To do this, we conducted multivariate classification analyses, which measure variable importance in a model's ability to correctly classify larvae as having died or survived. As only one out of 778 newt larvae died after being reared in control as both an embryo and larvae (see Results), we restricted our analyses to larvae reared in salt post-hatching. We used three validated classification procedures [57], logistic regression, Classification Trees [58] and Random Forests [57], [59], and in each case assessed variable importance by examining the relative classification performance of models incorporating or not incorporating key variables.

Specifically, we assessed the ability of the models to correctly classify larvae as having died (sensitivity). For the full model, we included all larval and embryonic variables of potential interest, including: larval treatment, embryonic treatment, length at hatching, developmental stage at hatching, and female identity. We then withdrew the larval treatment variable, and reassessed the model's sensitivity, withdrew all embryonic variables (leaving only larval treatment and individual female) and again reassessed the model's sensitivity, to assess the potential relative contribution of larval environment in predicting larval mortality. As well as assessing variable importance in this manner, all three classification methods also provide separate indicators of variable importance [57]. This is achieved through a variable importance plot in Random Forests, a classification plot in Classification Trees, and the variable with the largest Wald Chi-Square value in logistic regression. We chose the most important variable identified in each of these methods from the original full model, and reinserted it back into the model including only larval treatment and female identity, and assessed whether the inclusion of this identified variable increased model performance. Classification analyses were completed in SAS (logistic regression) and R (R Development Core Team, 2008, www.R-project.org) (Classification Trees and Random Forests). Finally, as length at hatching was identified as a key variable of importance in predicting larval mortality (see Results), we compared the mean length at hatching of larvae that died versus survived in each treatment using t-tests in SAS software version 9.3, with significance set at α = 0.05.

## Results

After 14 days, only one out of 778 larvae reared in control as both egg and larva (“control” treatment) died in this treatment, which was significantly fewer than in any other treatment (all *p*<0.001). The survival of the remaining larvae, all experiencing salts in their larval environment, was then compared. There was a significant effect of larval salt treatment on larval survival for both newts that were reared embryonically in salt (F_5,74_ = 16.54, *p*<0.0001) and control (F_5,73_ = 7.81, *p*<0.0001). For animals that were reared as eggs in salt and stayed in that salt as larvae, significantly more larvae died in low and medium MgCl_2_ than in those corresponding concentrations of NaCl (all Tukey adjusted multiple comparisons *p*<0.0001), with a similar percentage of larvae dying in high MgCl_2_ as high NaCl (Tukey adjusted *p* = 0.98). For animals that were reared as eggs in control and then transferred to salt as larvae, marginally more larvae died in low MgCl_2_ than low NaCl (Tukey adjusted *p* = 0.0698), and significantly more larvae died in medium and high MgCl_2_ than the corresponding concentrations of NaCl (Tukey adjusted *p*<0.02).

**Figure 2:**
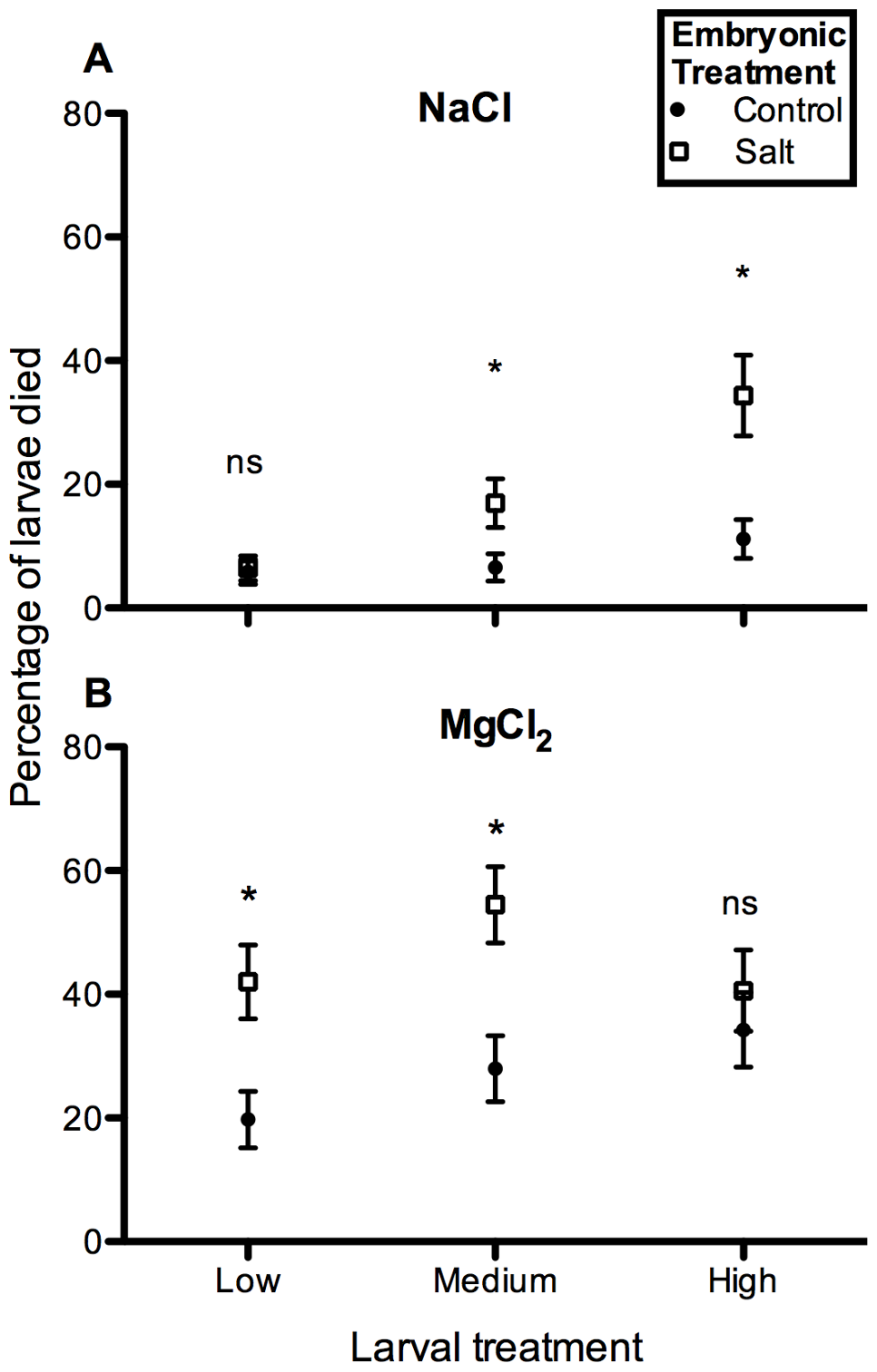
Percentage (mean ± SE) of larvae that died in each salt treatment. (A) NaCl, (B) MgCl_2_. Only 1 out of 778 larvae in Control died, and thus only results for mortality in salt treatments are shown. Direct comparisons are made between the mortality of larvae reared as embryos in salt (open squares) or control (closed circles). Asterisks indicate significant differences (Tukey-adjusted multiple comparisons) between the percentages of larvae died in each of these treatments (i.e., for the larval treatment Medium NaCl, significantly more larvae died when reared as eggs in that salt, than did larvae reared as eggs in control). “ns”  =  no significant difference between treatments.

Increased salt concentration, in both the embryonic and larval environments, generally resulted in increased larval mortality (with the exception of high MgCl_2_) (Fig. 2). For both salts, larval survival was significantly affected by embryonic environment (Table 1). For the majority of treatment levels, larvae that were reared as eggs in control solution survived significantly better than larvae that were reared as eggs in salt treatments (Fig. 2). For NaCl, both embryonic and larval treatments significantly affected survival of larvae in this salt, but for MgCl_2_, only embryonic treatment significantly explained larval survival (Table 1). There were no significant interacting effects of embryonic and larval environments on larval survival (Table 1).

**Table 1:** Effects of embryonic environment, larval environment, and their interaction on larval survival in NaCl and MgCl_2_.

Salt type	Embryonic environment			Larval environment			Embryonic x Larval environments		
	*F*	df (n,d)	*p*	*F*	df (n,d)	*p*	*F*	df (n,d)	*p*
NaCl	11.19	1,74	**0.0013**	9.61	2,74	**0.0002**	2.28	2,74	0.1095
MgCl_2_	18.34	1,73	**<0.0001**	2.22	2,73	0.1162	2.00	2,73	0.1429

Significant effects are listed in bold.

**Figure 3:**
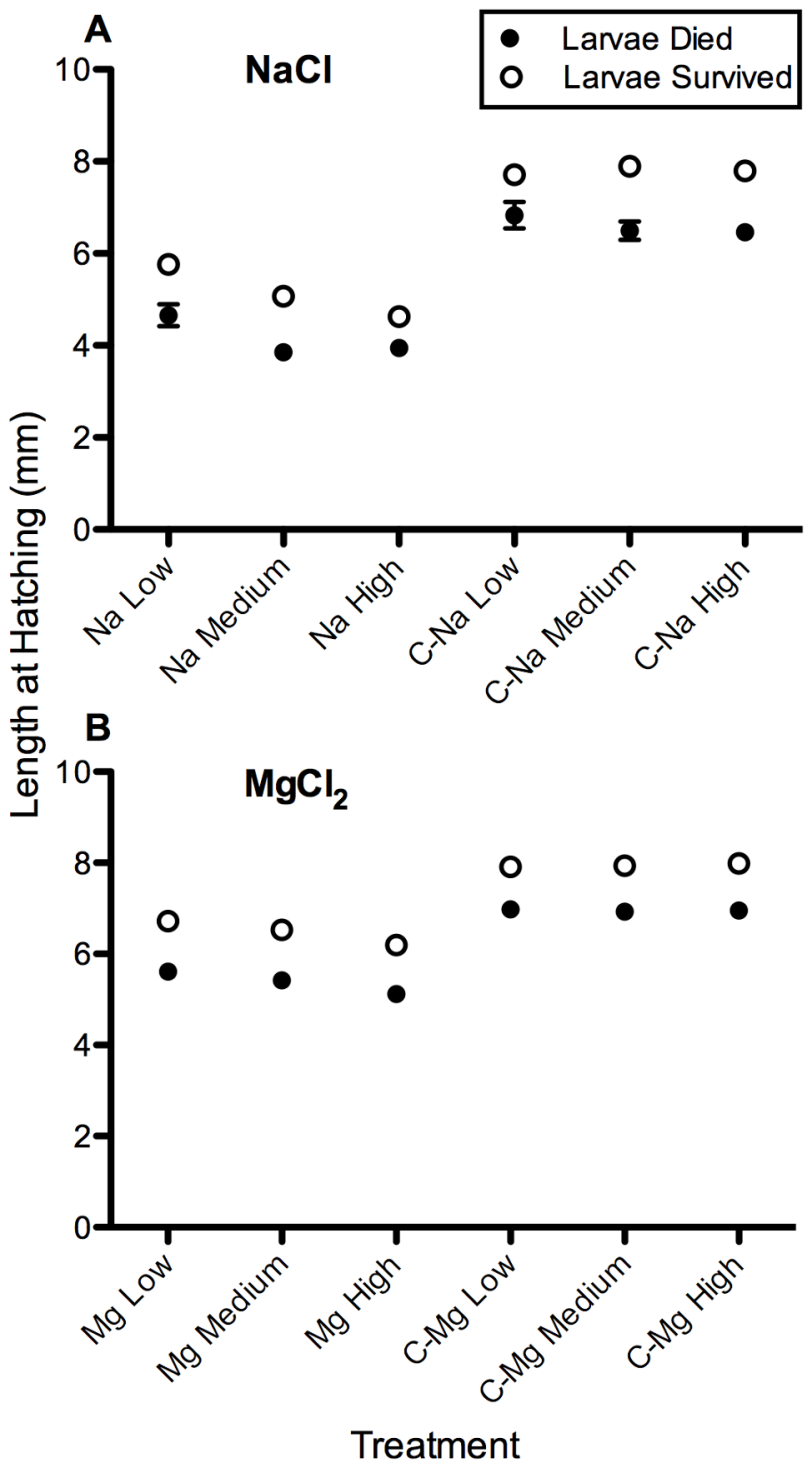
Mean (±SE) lengths at hatching (mm) of larvae that died (closed circles) or survived (open circles) in each salt treatment. (A) NaCl, (B) MgCl_2_. In all treatments, larvae that survived averaged larger at hatching than those that died (all t-tests, *p*<0.01). See [44] for full results on length at hatching.

Eggs that were reared in salt water resulted in smaller larvae at hatching than those reared in control [44]. Classification analyses with three different methods all revealed length at hatching as the consistently most important variable in determining larval survival (Table 2), further strengthening the evidence of the importance of embryonic environment on survival post-hatching. Although Classification Trees and Random Forests had better sensitivity than logistic regression (as was expected [57]), the ability of models, using any of the classification methods, to correctly classify larvae as having died declined dramatically with the exclusion of embryonic variables (i.e., larval treatment and female identity alone was a very poor classifier of larval survival), but recovered substantially with the re-inclusion of length at hatching as a predictor variable (Table 2), further identifying it as a critical variable for predicting larval survival. Larvae that survived, in each of the treatments, were significantly larger at hatching, on average, than larvae that died (Fig. 3; for all t-tests, *p*<0.01).

**Table 2:** Classification analyses for predicting whether or not newt larvae died (“sensitivity”), for data excluding control data (i.e., only newts in salt as larvae).

Model	Classification method	Model sensitivity (%) (percent larvae correctly classified as having died)	Change in model sensitivity from full model sensitivity (%)	Most important variable identified
Full (Larval Treatment, Egg Treatment, Length & Stage at Hatching, Female)	Logistic Regression	47.70	.	Length at Hatching
	Classification Trees	69.65	.	Length at Hatching
	Random Forests	64.78	.	Length at Hatching
Just Embryonic Variables (Egg Treatment, Length & Stage at Hatching, Female) (not Larval Treatment)	Logistic Regression	39.82	−7.88	Egg Treatment
	Classification Trees	64.39	−5.26	Length at Hatching
	Random Forests	59.26	−5.52	Length at Hatching
Just Larval Treatment and Female	Logistic Regression	13.67	−34.03	Larval Treatment
	Classification Trees	19.84	−49.81	Larval Treatment
	Random Forests	14.32	−50.46	Larval Treatment
Just Larval Treatment, Female, & Length at Hatching	Logistic Regression	44.42	−3.28	Length at Hatching
	Classification Trees	61.76	−7.89	Length at Hatching
	Random Forests	52.56	−12.22	Length at Hatching

Three multivariate classification methods were utilized (logistic regression, Classification Trees, and Random Forests) to determine the most important variables predicting larval survival in salt. See Methods and Results for more details regarding these analyses and their interpretation.

## Discussion

**Figure 4:**
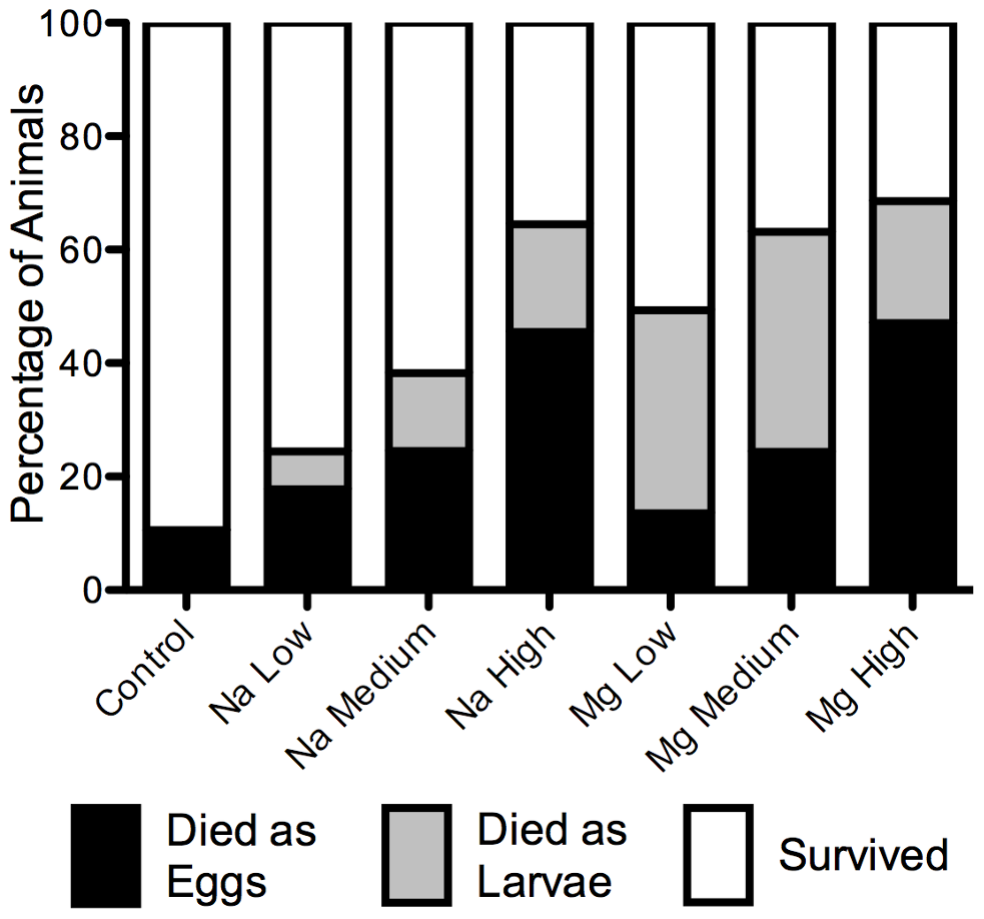
Mortality of newt eggs (black bars) and larvae (grey bars) in each salt treatment. This figure shows only larvae that were reared as both eggs and larvae in salt. The percentage of individuals that survived in each treatment is indicated in white. All percentages are calculated based on the total number of eggs that started in each treatment (Control  = 2577, low NaCl  = 363, medium NaCl = 366, high NaCl = 345, low MgCl_2_ = 369, medium MgCl_2_ = 369, high MgCl_2_ = 354; [44]), some of which either died (black bars), or survived to hatching and were reared in salt, where they either died (grey bars) or survived (white bars).

Developmental and evolutionary history each significantly affected the survival of newt larvae in salts, and thus the importance of both hypotheses was supported. Eggs appear to be a critical life history stage for this amphibian in osmotically stressful environments. Animals that were exposed to salt as embryos and survived hatched at a smaller size than animals that did not experience embryonic salinity. Stunting of embryonic growth put amphibian larvae at greater risk for salt-induced mortality (Table 2). However, our results show that it is also important to understand the evolutionary history an organism has with a stressor. Even though there was no difference in egg mortality between embryos reared in NaCl or MgCl_2_ [44], more larvae died in MgCl_2_ than in NaCl (Fig. 4). While newt larvae have evolved with natural sources of NaCl in their environment, which they can osmoregulate, such common regulation of MgCl_2_ does not appear to have evolved. Understanding this evolutionary history, as well as parsing critical life history stages is imperative to understand the effects of stressors on the life history of an organism.

The majority of organisms have complex life cycles, and the experiences of one life stage can have profound impacts on those in subsequent stages [1] (Table S1). Embryonic salinity is known to affect the post-hatching survival, growth and development of marine and estuarine invertebrates, such as barnacles [23], crabs [15], [25], [27], horseshoe crabs [26] and tunicates [28]. While all life history stages of amphibians have, individually, repeatedly been found to be extremely sensitive to salt [21], [47], [54], [55], [60]–[68], with a few notable exceptions such as *Fejervarya cancrivora* [69], [70], the relative sensitivity of each life history stage, and potential down-stream effects of salinity from one stage to the next, have been less studied. In one of the only other studies on amphibians to examine embryonic carry-over effects of salinity, frog larvae (*Lithobates sylvaticus*) reared in salt water (NaCl-based) as eggs had reduced survival in salt compared to larvae that were reared in freshwater as eggs [47]. This study also found that growth and development of larvae that survived was depressed in those animals reared embryonically in salt, also suggesting carry-over effects of embryonic exposure to salt [47]. Snodgrass et al [21] also found that *Bufo americanus* toadlets exposed to stormwater pond sediment (which had an increased conductivity mainly due to road deicing salt) as embryos were smaller at metamorphosis than embryos and larvae exposed to freshwater (although the relative effects of embryonic vs. larval exposure were not separated). Other studies have also shown potential carry-over effects of larval salinity exposure on metamorphic traits important for adult fitness [37], [60]. These results all clearly show that Qiu and Qian's [23] statement regarding marine invertebrates, that “osmotic stress experienced in one life-stage can be passed over to the next life-stage”, can apply to freshwater vertebrates as well.

This pattern of decreased post-hatching survival as a consequence of embryonic exposure has also been found in amphibians in response to other stressors, such as nitrite [71] and pesticides [22]. Thus, studies that do not examine effects at each life history stage and do not consider the potential for cascading effects across stages may seriously underestimate the cumulative effects of exposure to stressors [19], [71]–[73].

One of the primary ways that osmotic stress affects the embryonic stage to influence post-hatching survival is through the retardation of growth and development. Newt eggs that were reared in salt water hatched sooner, smaller and less developed than newts reared in a freshwater control [44], and this resulting reduced length at hatching appears to be the single most important variable in predicting next-stage (larval) survival in salt water (Table 2). Size at hatching/birth is well known to have important implications on larval, juvenile, and adult health and survival in a wide variety of taxa, ranging from sea snails [74] and bryozoans [12], to birds [75] and humans [7]. Furthermore, this link between size and fitness has been identified as key to life history theory [12]. Among amphibians, hatching early, smaller and less developed, is known to affect larval survival, the onset of feeding competence, competitive and predatory interactions, and larval growth rate and timing of metamorphosis [6], [76]–[80]. Similar to our findings, small, less developed amphibian larvae are more susceptible to pollutants than are large larvae [81], [82]. Smaller larval rough-skinned newts are also more vulnerable to be injured and die in predatory encounters with dragonfly nymphs [83]. Thus, even if smaller hatchlings are able to survive short-term in osmotically stressful environments (which seems unlikely from our results (Fig. 3)), or even if compensatory growth occurred later in development, a host of other fitness consequences of this initial stunted embryonic growth and development are still likely later in life [84], further emphasizing the importance of the embryonic environment for life-time fitness.

While the effects of the two salt types were not significantly different on embryonic survival [44], there were differences in the larval stage, whereby MgCl_2_ had relatively greater effects on survival (Fig. 4). This is in spite of the fact that embryos actually hatched slightly larger at MgCl_2_ than at NaCl [44]. Although most amphibian eggs, like those of many other aquatic organisms [85], have little means of osmoregulating at the salt concentrations used in this study [37], [63], [86], and thus the effects of NaCl and MgCl_2_ at this life history stage are equally destructive (any affect of evolutionary history is minimized in the absence of regulatory ability), amphibian larvae have evolved to osmoregulate Na^+^ and Cl^−^ ion concentrations in their body through the use of integumental and gill Na^+^ pumps [31], [87]–[90]. Larvae have not evolved this same ability to regulate Mg^2+^ ions, however, and thus larvae in NaCl were able to attempt osmoregulation to survive in this solution whereas larvae in MgCl_2_ were not. In addition to lacking this evolutionary history of osmoregulation, Mg^2+^ has also been shown to be inhibitory to important osmoregulatory skin ion pump functioning in other amphibian larvae [91]. Whereas the effects of NaCl on larvae act in a typical dose-response fashion (Fig. 2a), it appears that any concentration of MgCl_2_ is detrimental to larvae (Fig. 2b), as they have less means to regulate it. This may explain why larval salinity concentration significantly influences larval survival for animals in NaCl, but not those in MgCl_2_ (Table 1). The fact that Mg High had lower mortality than Mg Low or Medium (Fig. 2b) may be due to a number of possible reasons, including hormesis [92]. In the only other studies on the effects of MgCl_2_ on amphibian larvae to date, both Dougherty and Smith [62] and Harless et al [54] also found that this emerging deicing salt was more toxic to frog tadpoles than NaCl. Magnesium chloride may in fact, be more toxic than NaCl to life in general, as studies have found that otherwise salt-tolerant plants [93], [94] and archaea [95] are often intolerant of MgCl_2_, and the threshold for biological processes in MgCl_2_ is lower than other salts, including NaCl [95]. These results make sense from an evolutionary perspective, given the small quantities of Mg^2+^ generally found in most aquatic ecosystems, relative to the higher quantities of Na^+^ found in precipitation and the ocean [32], and thus many organisms may not have an evolutionary history of regulating Mg^2+^ in high concentrations in their environment.

Vulnerability of a particular life history stage can be described as the ability of that life history stage to regulate the stressor in question. Using this criterion, it appears that eggs are the most vulnerable life history stage to salts overall in amphibians (this study; [61], [65], [66], [96]) and effects on embryonic development at this stage have profound survival consequences in later life history stages, even possibly affecting population viability indirectly through influencing post-embryonic (larval) mortality [97]. Similarly, amphibian larvae cannot successfully osmoregulate in MgCl_2_, and thus all life history stages are particularly vulnerable to this evolutionarily novel but emerging deicing agent, which is now the second most commonly used road deicer in North America [33].

### Conclusions

Understanding the evolutionary history of an organism with its stressor, and the differential sensitivity of life history stages to that stressor are critical in assessing the vulnerability of organisms to stressful environments. It is now apparent that embryonic exposure to a stressor can have profound implications on the post-hatching survival and fitness of organisms in practically all animal taxa (Table S1), through influencing growth and development in this critical life history stage. In post-hatching individuals, however, even the largest, best-developed organism can only successfully deal with stressors that they have evolved to regulate. As the world of these organisms becomes increasingly impacted by anthropogenic factors, understanding this evolutionary history and its survival implications at and across different life history stages will be critical for the future conservation of animals in increasingly stressful environments.

## Supporting Information

Table S1 Animal phyla where components of the embryonic environment have been demonstrated to have significant carry-over effects post-hatching. This list is not exhaustive, but is representative of the diversity and breadth of this phenomenon throughout the animal kingdom.
(DOCX)
