# Supplementary material for: Developmental and Evolutionary History Affect Survival in Stressful Environments
Source: PLoS One. 2014 Apr 18;9(4):e95174. doi: 10.1371/journal.pone.0095174 (PMC3991610; doi:10.1371/journal.pone.0095174)
Supplement: Table S1 — Animal phyla where components of the embryonic environment have been demonstrated to have significant carry-over effects post-hatching. This list is not exhaustive, but is representative of the diversity and breadth of this phenomenon throughout the animal kingdom. (DOCX) [file pone.0095174.s001.docx]

**Supporting Information**

***For Hopkins, G.R., Brodie, Jr., E.D., and S.S. French. 2013. Developmental and evolutionary history affect survival in stressful environments. PLoS ONE***

**Table S1.** Animal phyla where components of the embryonic environment have been demonstrated to have significant carry-over effects post-hatching. This list is not exhaustive, but is representative of the diversity and breadth of this phenomenon throughout the animal kingdom.

| Phyla  Subphyla: Class | Examples | Traits / endpoints affected by embryonic environment | Component of embryonic environment | References |
| --- | --- | --- | --- | --- |
|  |  |  |  |  |
| Bryozoa |  |  |  |  |
| Gymnolaemata | Bryozoans | Larval size, leading to larval survival, growth rate, timing and rate of reproduction, size of subsequent embryos and larvae | Colony size | [1] |
|  |  |  |  |  |
| Arthropoda |  |  |  |  |
| Crustacea : Maxillopeda | Barnacles | Larval survival; Time to metamorphosis | Salinity | [2] |
|  |  |  |  |  |
| Crustacea : Malacostraca | Crabs | Larval survival, body nutritional content, development, growth, starvation tolerance; size at metamorphosis; juvenile size; larval osmoregulatory ability; | Salinity;  Temperature | [3-5, reviewed by 6] |
|  |  |  |  |  |
| Hexapoda : Insecta | Butterflies, Beetles | Larval size, energy content, body nutritional composition; size at hatching; adult size | Temperature | [7, 8] |
|  |  |  |  |  |
| Chelicerata : Merostomata | Horshoe Crabs | Length of larval period (molting to first juvenile instar) | Salinity | [9] |
|  |  |  |  |  |
| Mollusca |  |  |  |  |
| Gastropoda | Sea Snail | Hatchling size, leading to juvenile and adult survival, growth, time to maturity | Brood size | [10] |
|  | Freshwater Snail | Juvenile predator avoidance behaviour | Temperature | [11] |
|  |  |  |  |  |
| Echinodermata |  |  |  |  |
| Asterozoa | Seastar | Larval survival; larval growth; successful metamorphosis | Maternal nutrition | [12] |
|  |  |  |  |  |
| Echinozoa | Sea urchins | Larval survival; larval growth; successful metamorphosis; larval settlement success | Maternal nutrition;  CO_2_ acidity | [12, 13] |
|  |  |  |  |  |
| Chordata |  |  |  |  |
| Urochordata : Ascidiacea | Tunicates (sea-squirts) | Adult functional development | Salinity; Temperature | [14] |
|  |  |  |  |  |
| Vertebrata : Actinopterygii | Ray-finned bony fishes | Juvenile body size and shape; larval prey-capture ability; larval growth rate and development; juvenile growth rate; larval and juvenile muscle growth and development; larval gut development; Survival up to 2 years post-hatching; Adult cardiac morphology, aerobic capacity (swimming performance); juvenile antipredator / predator avoidance behaviour; 1 year post-hatching humoral immunosuppression; larval survival | Temperature;  Methylmercury;  Acidity;  Crude oil; Cadmium;  Hypoxia;  Pesticide (DDT) metabolites; Aluminum | [15-26] |
|  |  |  |  |  |
| Vertebrata : Amphibia | Frogs, toads, salamanders, newts | Larval survival; predator avoidance behaviour; predation survival; larval growth; larval insecticide susceptibility; larval feeding rate; larval size and development 1 month post-hatching; Size at metamorphosis; Juvenile behaviour and desiccation risk up to 8 months post-metamorphosis; Juvenile survival; Time to metamorphosis; swimming performance; size-independent morphology; larval activity level; Post-metamorphic (juvenile) morphology | Salinity; Stormwater sediments (increased conductivity); Temperature; UV-B radiation; Pesticide; PCBs; Herbicide; Nitrite; Acidity (pH); Hydration; Conspecific chemical alarm cues; Predatory chemical cues; Predator presence; | This study, [27-48] |
|  |  |  |  |  |
| Vertebrata : Sauropsida (“Reptilia”) | Turtles, Lizards, Snakes, Crocodilians | Hatchling size; Juvenile growth rate; Size at up to 2 years post-hatching; Sex; Escape behaviour; Juvenile survival up to 2 years; Juvenile habitat temperature choice and thermoregulation; Sprint speed; Juvenile activity level, ability to move, climb, avoid predators, shedding time and drinking speed; antipredator behaviour; body contraction strength; swimming speed | Temperature;  Herbicide; Hypoxia | [49-57] |
|  |  |  |  |  |
| Vertebrata : Sauropsida (“Aves”) | Birds | Juvenile growth, body condition, immunocompetence, metabolic costs of thermoregulation; Recruitment probability; Fecundity; juvenile morphology; | Temperature; Maternal Nutrition | [58-63] |
|  |  |  |  |  |
| Vertebrata : Mammalia | Rats, Humans, Moose, Hamsters | Sedentary behaviour; risk of cardiac disease; type 2 diabetes; adult body mass; adult offspring endocrine profiles and antipredator behaviour | Physical size of womb;  Maternal nutrition; Climatic conditions (temperature, snow depth); Maternal immune activation | [63-68] |

**References:**

1. Marshall DJ, Bolton TF, Keough MJ (2003) Offspring size affects the post-metamorphic performance of a colonial marine invertebrate. Ecology 84: 3131-3137.

2. Qiu J-W, Qian P-Y (1999) Tolerance of the barnacle *Balanus amphitrite amphitrite* to salinity and temperature stress: effects of previous experience. Marine Ecology Progress Series 188: 123-132.

3. Giménez L (2002) Effects of prehatching salinity and initial larval biomass on survival and duration of development in the zoea 1 of the estuarine crab, *Chasmagnathus granulata*, under nutritional stress. Journal of Experimental Marine Biology and Ecology 270: 93-110.

4. Giménez L, Anger K (2003) Larval performance in an estuarine crab, *Chasmagnathus granulata*, is a consequence of both larval and embryonic experience. Marine Ecology Progress Series 249: 251-264.

5. Charmantier G, Giménez L, Charmantier-Daures M, Anger K (2002) Ontogeny of osmoregulation, physiological plasticity and larval export strategy in the grapsid crab *Chasmagnathus granulata* (Crustacea, Decapoda). Marine Ecology Progress Series 229: 185-194.

6. Giménez L (2006) Phenotypic links in complex life cycles: conclusions from studies with decapod crustaceans. Integrative and Comparative Biology 46: 615-622.

7. Geister TL, Lorenz MW, Hoffman KH, Fischer K (2009) Energetics of embryonic development: effects of temperature on egg and hatchling composition in a butterfly. Journal of Comparative Physiology B 179: 87-98.

8. Ernsting G, Isaaks JA (1997) Effects of temperature and season on egg size, hatchling size and adult size in *Notiophilus biguttatus*. Ecological Entomology 22: 32-40.

9. Ehlinger GS, Tankersley RA (2004) Survival and development of horsehoe crab (*Limulus polyphemus*) embryos and larvae in hypersaline conditions. Biological Bulletin 206: 87-94.

10. Moran AL, Emlet RB (2001) Offspring size and performance in variable environments: field studies on a marine snail. Ecology 82: 1597-1612.

11. Dalesman S, Rundle SD (2010) Influence of rearing and experimental temperatures on predator avoidance behaviour in a freshwater pulmonate snail. Freshwater Biology 55: 2107-2113.

12. George SB (1995) Echinoderm egg and larval quality as a function of adult nutritional state. Oceanologica Acta 19: 297-308.

13. Dupont S, Dorey N, Stumpp M, Melzner F, Thorndyke M (2012) Long-term and trans-life-cycle effects of exposure to ocean acidification in the green sea urchin *Strongylocentrotus droebachiensis*. Marine Biology.

14. Thiyagarajan V, Qian P-Y (2003) Effect of temperature, salinity and delayed attachment on development of the solitary ascidian *Styela plicata* (Lesueur). Journal of Experimental Marine Biology and Ecology 290: 133-146.

15. Eriksen MS, Bakken M, Espmark A, Braastad BO, Salte R (2006) Prespawning stress in farmed Atlantic salmon *Salmo salar*: maternal cortisol exposure and hyperthermia during embryonic development affect offspring survival, growth and incidience of malformations. Journal of Fish Biology 69: 114-129.

16. Georgakopoulou E, Sfakianakis DG, Kouttouki S, Divanch P, Kentouri M, et al. (2007) The influence of temperature during early life on phenotypic expression at later ontogenetic stages in sea bass. Journal of Fish Biology 70: 278-291.

17. Weis JS, Weis JS (1995) Effects of embryonic exposure to methylmercury on larval prey-capture ability in the mummichog, *Fundulus heteroclitus*. Environmental Toxicology and Chemistry 14: 153-156.

18. Heintz RA, Rice SD, Wertheimer AC, Bradshaw RF, Thrower FP, et al. (2000) Delayed effects on growth and marine survival of pink salmon *Oncorhynchus gorbuscha* after exposure to crude oil during embryonic development. Marine Ecology Progress Series 208: 205-216.

19. Hicken CE, Linbo TL, Baldwin DH, Willis ML, Myers MS, et al. (2011) Sublethal exposure to crude oil during embryonic development alters cardiac morphology and reduces aerobic capacity in adult fish. Proceedings of the National Academy of Sciences of the United States of Amerca (PNAS) 108: 7086-7090.

20. Johnston IA, Cole NJ, Abercromby M, Vieira VLA (1998) Embryonic temperature modulates muscle growth characteristics in larval and juvenile herring. Journal of Experimental Biology 201: 623-646.

21. Kusch RC, Krone PH, Chivers DP (2007) Chronic exposure to low concentartions of waterborne cadmium during embryonic and larval development results in the long-term hindrance of antipredator behavior in zebrafish. Environmental Toxicology and Chemistry 27: 705-710.

22. Martell DJ, Kieffer JD, Trippel EA (2005) Effects of temperature during early life history on embryonic and larval development and growth in haddock. Journal of Fish Biology 66: 1558-1575.

23. Martell DJ, Kieffer JD, Trippel EA (2006) Effects of the embryonic thermal environment on haddock (*Melanogrammus aeglefinus*) developmental trajectories through exogenous feeding stages. Marine Biology 149: 177-187.

24. Roussel J-M (2007) Carry-over effects in brown trout (*Salmo trutta*): hypoxia on embryos impairs predator avoidance by alevins in experimental channels. Canadian Journal of Fisheries and Aquatic Science 64: 786-792.

25. Milston RH, Fitzpatrick MS, Vella AT, Clements S, Gundersen D, et al. (2003) Short-term exposure of chinook salmon (*Oncoryhnchus tshawytscha*) to *o,p*'-DDE or DMSO during early life-history stages causes long-term humoral immunosuppression. Environmental Health Perspectives 111: 1601-1607.

26. McCormick JH, Jensen KM, Anderson LE (1988) Chronic effects of low pH and elevated alumninum on survival, maturation, spawning and embryo-larval development of the fathead minnow in soft water. Water, Air, and Soil Pollution 43.

27. Petranka JW, Doyle EJ (2010) Effects of road salts on the composition of seasonal pond communities: can the use of road salts enhance mosquito recruitment? Aquatic Ecology 44: 155-166.

28. Snodgrass JW, Casey RE, Joseph D, Simon JA (2008) Microcosm investigations of stormwater pond sediment toxicity to embryonic and larval amphibians: variation in sensitivity among species. Environmental Pollution 154: 291-297.

29. Broomhall SD (2004) Egg temperature modifies predator avoidance and the effects of the insecticide endosulfan on tadpoles of an Australian frog. Journal of Applied Ecology 41: 105-113.

30. Belden LK, Blaustein AR (2002) Exposure of red-legged frog embryos to ambient UV-B radiation in the field negatively affects larval growth and development. Oecologia 130: 551-554.

31. Bridges CM (2000) Long-term effects of pesticide exposure at various life stages of the sourthern leopard frog (*Rana sphenocephala*). Archives of Environmental Contamination and Toxicology 39: 91-96.

32. Gutleb AC, Appelman J, Bronkhorst MC, van den Berg JHJ, Spenkelink A, et al. (1999) Delayed effects of pre- and early-life time exposure to polychlorinated biphenyls on tadpoles of two amphibian species (*Xenopus laevis* and *Rana temporaria*). Environmental Toxicology and Pharmacology 8: 1-14.

33. Rohr JR, Palmer BD (2005) Aquatic herbicide exposure increases salamander desiccation risk eight months later in a terrestrial environment. Environmental Toxicology and Chemistry 24: 1253-1258.

34. Rohr JR, Sager T, Sesterhenn TM, Palmer BD (2006) Exposure, postexposure, and density-mediated effects of atrazine on amphibians: breaking down net effects into their parts. Environmental Health Perspectives 114: 46-50.

35. Greulich K, Pflugmacher S (2003) Differences in susceptibility of various life stages of amphibians to pesticide exposure. Aquatic Toxicology 65: 329-336.

36. Griffis-Kyle KL (2005) Ontogenetic delays in effects of nitrite exposure on tiger salamanders (*Ambystoma tigrinum*) and wood frogs (*Rana sylvatica*). Environmental Toxicology and Chemistry 24: 1523-1527.

37. Räsänen K, Laurila A, Merilä J (2002) Carry-over effects of embryonic acid conditions on development and growth of *Rana temporaria* tadpoles. Freshwater Biology 47: 19-30.

38. Smith GR, Waters MA, Rettig JE (2000) Consequences of embryonic UV-B exposure for embryos and tadpoles of the plains leopard frog. Conservation Biology 14: 1903-1907.

39. Pahkala M, Laurila A, Merilä J (2001) Carry-over effects of ultraviolet-B radiation on larval fitness in *Rana temporaria*. Proceedings of the Royal Society B 268: 1699-1725.

40. Watkins TB, Vraspir J (2006) Both incubation temperature and posthatching temperature affect swimming performance and morphology of wood frog tadpoles (*Rana sylvatica*). Physiological and Biochemical Zoology 79: 140-149.

41. Orizaola G, Dahl E, Laurila A (2010) Compensating for delayed hatching across consecutive life-history stages in an amphibian. Oikos 119: 980-987.

42. Touchon JC, Warkentin KM (2010) Short- and long-term effects of the abiotic egg environment on viability, development and vulnerability to predators of a Neotropical anuran. Functional Ecology 24: 566-575.

43. Mandrillon A-L, Saglio P (2009) Effects of single and combined embryonic exposures to herbicide and conspecific chemical alarm cues on hatching and larval traits in the common frog (*Rana temporaria*). Archives of Environmental Contamination and Toxicology 56: 566-576.

44. Smith MJ, Drew M, Peebles M, Summers K (2005) Predator cues during the egg stage affect larval development in the gray tree frog (*Hyla versicolor*) (Anura: Hylidae). Copeia 2005: 169-173.

45. Orizaola G, Braña F (2005) Plasticity in newt metamorphosis: the effect of predation at embryonic and larval stages. Freshwater Biology 50: 438-446.

46. Saglio P, Mandrillon A-L (2006) Embryonic experience to predation risk affects tadpoles of the common frog (*Rana temporaria*). Archiv fuer Hydrobiologie 166: 502-523.

47. Laurila A, Pakkasmaa S, Crochet P-A, Merilä J (2002) Predator-induced plasticity in early life history and morphology in two anuran amphibians. Oecologia 132: 524-530.

48. Mathis A, Ferrari MCO, Windel N, Messier F, Chivers DP (2008) Learning by embryos and the ghost of predation future. Proceedings of the Royal Society B 275: 2603-2607.

49. Brooks RJ, Bobyn ML, Galbraith DA, Layfield JA, Nancekivell EG (1991) Maternal and environmental influences on growth and survival of embryonic and hatchling snapping turtles (*Chelydra serpentina*). Canadian Journal of Zoology 69: 2667-2676.

50. McKnight CM, Gutzke WHN (1993) Effects of the embryonic environment and of hatchling housing conditions on growth of young snapping turtles. Copeia 1993: 475-482.

51. Neuman-Lee LA, Janzen FJ (2011) Atrazine exposure impacts behavior and survivorship or neonatal turtles. Herpetologica 67: 23-31.

52. O'Steen S (1998) Embryonic temperature influences juvenile temperature choice and growth rate in snapping turtles *Chelydra serpentina*. Journal of Experimental Biology 201: 439-449.

53. Van Damme R, Bauwens D, Braña F, Verheyen RF (1992) Incubation temperature differentially affects hatching time, egg survival, and hatchling performance in the lizard *Podarcis muralis*. Herpetologica 48: 220-228.

54. Burger J (1989) Incubation temperature has long-term effects on behaviour of young pine snakes (*Pituophis melanoleucus*). Behavioral Ecology and Sociobiology 24: 201-207.

55. Webb GJW, Cooper-Preston H (1989) Effects of incubation temperature on crocodiles and the evolution of reptilian oviparity. American Zoologist 29: 953-971.

56. Stahlschmidt ZR, DeNardo DF (2009) Obligate costs of parental care to offspring: egg brooding-induced hypoxia creates smaller, slower and weaker python offspring. Biological Journal of the Linnean Society 98: 414-421.

57. Elphick MJ, Shine R (1998) Longterm effects of incubation temperatures on the morphology and locomotor performance of hatchling lizards (*Bassiana duperreyi*, Scincidae). Biological Journal of the Linnean Society 63: 429-447.

58. DuRant SE, Hopkins WA, Hawley DM, Hepp GR (2011) Incubation temperature affects multiple measures of immunocompetence in young wood ducks (*Aix sponsa*). Biology Letters 8: 108-111.

59. DuRant SE, Hopkins WA, Wilson AF, Hepp GR (2011) Incubation temperature affects the metabolic cost of thermoregulation in a young precocial bird. Functional Ecology 26: 416-422.

60. Spear L, Nur N (1994) Brood size, hatching order and hatching date: effects on four life-history stages from hatching to recruitment in western gulls. Journal of Animal Ecology 63: 283-298.

61. Gorman HE, Nager RG (2004) Prenatal developmental conditions have long-term effects on offpsring fecundity. Proceedings of the Royal Society B 271: 1923-1928.

62. Monaghan P (2008) Early growth conditions, phenotypic development and environmental change. Philosophical Transactions of the Royal Society B 363: 1635-1645.

63. Lindström J (1999) Early development and fitness in birds and mammals. Trends in Ecology and Evolution 14: 343-348.

64. Vickers MH, Breier BH, McCarthy D, Gluckman PD (2003) Sedentary behavior during postnatal life is determined by the prenatal environment and exacerbated by postnatal hypercaloric nutrition. American Journal of Physiology - Regulatory, Integrative and Comparative Physiology 285: R271-R273.

65. Gluckman PD, Hanson MA, Beedle AS (2007) Early life events and their consequences for later disease: a life history and evolutionary perspective. American Journal of Human Biology 19: 1-19.

66. Barker DJP (2006) Adult consequences of fetal growth restriction. Clinical obstetrics and gynecology 49: 270-283.

67. Solberg EJ, Loison A, Gaillard J-M, Heim M (2004) Lasting effects of conditions at birth on moose body mass. Ecography 27: 677-687.

68. French SS, Chester EM, Demas GE (2013) Maternal immune activation affects litter success, size and neuroendocrine responses related to behavior in adult offspring. Physiology and Behavior 119: 175-184.
